# Anthropometry, Body Composition and Resting Energy Expenditure in Human

**DOI:** 10.3390/nu11081891

**Published:** 2019-08-14

**Authors:** Josep A. Tur, Maria del Mar Bibiloni

**Affiliations:** Research Group on Community Nutrition and Oxidative Stress, University of Balearic Islands, IDISBA & CIBEROBN (Physiopathology of Obesity and Nutrition), 07122 Palma de Mallorca, Spain

**Keywords:** anthropometry, body composition, body mass index, free fat mass, body fat, nutritional status, dietary influences, lifestyle outcomes

Anthropometry (from the Greek *anthropos*: human, and *metron*: measure) refers to the systematic collection and correlation of measurements of human individuals, including the systematic measurement of the physical characteristics of the human body, primarily body weight, body size, and shape. Today, anthropometry includes single, portable, easily applicable, non-invasive, and inexpensive techniques to assess size and composition of the human body, reflecting health and nutritional status [1]. Today, anthropometric and body composition indicators are useful to predict the development of noncommunicable diseases, like diabetesor cardiovascular diseases [2,3], but it is also useful to assess relationships with physical condition and an active/inactive lifestyle, as well as thedecline of physical ability and sarcopenia incidence [4]. Therefore, anthropometric measurements are needed as part of methods to develop strategies for early identification of decline in physical condition and appropriate interventions to avoid physical impairments, and to promote quality of life.

Resting energy expenditure (REE) is the energy expenditure of an individual who is not fasting and is the number of calories required for a 24 h period by the body during a non-active period [5]. REE usually accounts for more than 60% of the total energy expenditure and is directly related to the amount of fat-free mass, which is more active metabolically than fat mass [6].The REE is useful to avoid or prevent underfeeding and/or overfeeding of individuals, especially in clinical care, but it also crucial to establish reachable goals for dietary and exercise interventions. REE can be estimated by numerous published formulas. Since the most used Harris–Benedict equation in 1918 [7], nearly 200 published REE formulas have been published dealing with various conditions [8], and the body composition is relevant to assess the validity of REE equations, which mainly depends on gender, age, and weight status [9].

The reliability and precision of body compartment measurements over a range of BMIs have been examinedby means of several techniques. Dual X-ray absorptiometry (DXA) and bioelectrical impedance devices (BIA) are the most used and precise methods. However, BIA lightly underestimated fat mass and overestimated fat-free mass and visceral adipose tissue compared to DXA [10,11]. However, BIA proved to be useful to measure changes in fat mass, body fat, total and skeletal muscle mass, ratio of lower extremity muscle mass, and ratio of upper extremity muscle mass to body weight in gastrectomized patients [12]. Simple anthropometric measurements, like waist circumference [10,13], are also useful and very informative, and BMI and body weight are still the most used parameters, in both clinical and epidemiological studies. In this way, studies on dietary and lifestyle intervention have used anthropometric, body weight, and body composition parameters as the basis of their assessment [14,15,16].

Data on nutritional status of human populations are periodically needed, as well as their relationships with anthropometry, body composition, body image, and energy expenditure, and also with healthy lifestyle outcomes. All these parameters contribute jointly to give a complete knowledge on dietary and lifestyle habits, and hence how to proceed to improve it in order to enjoy an optimal healthy status. Therefore, this Special Issue of *Nutrients* was designed and developed.
